# Capacitive monitoring system for real‐time respiratory motion monitoring during radiation therapy

**DOI:** 10.1002/acm2.12958

**Published:** 2020-07-09

**Authors:** Parisa Sadeghi, Kathryn Moran, James L. Robar

**Affiliations:** ^1^ Department of Physics and Atmospheric Science Dalhousie University Halifax Nova Scotia Canada; ^2^ Department of Radiation Oncology Dalhousie University Halifax Nova Scotia Canada; ^3^ Department of Radiation Therapy Services Queen Elizabeth II Health Sciences Centre Halifax Nova Scotia Canada

**Keywords:** capacitive monitoring system, motion detection, respiratory motion

## Abstract

**Summary:**

This work introduces a novel capacitive‐sensing technology capable of detecting respiratory motion with high temporal frequency (200 Hz). The system does not require contact with the patient and has the capacity to sense motion through clothing or plastic immobilization devices.

**Abstract:**

**Purpose:**

This work presents and evaluates a novel capacitive monitoring system (CMS) technology for continuous detection of respiratory motion during radiation therapy. This modular system provides real‐time motion monitoring without any contact with the patient, ionizing radiation, or surrogates such as reflective markers on the skin.

**Materials and methods:**

The novel prototype features an array of capacitive detectors that are sensitive to the position of the body and capable of high temporal frequency readout. Performance of this system was investigated in comparison to the RPM infrared (IR) monitoring system (Varian Medical Systems). The prototype included three (5 cm × 10 cm) capacitive copper sensors in one plane, located at a distance of 8–10 cm from the volunteer. Capacitive measurements were acquired for central and lateral‐to‐central locations during chest free‐breathing and abdominal breathing. The RPM IR data were acquired with the reflector block at corresponding positions simultaneously. The system was also tested during deep inspiration and expiration breath‐hold maneuvers.

**Results:**

Capacitive monitoring system data demonstrate close agreement with the RPM status quo at all locations examined. Cross‐correlation analysis on RPM and CMS data showed an average absolute lag of 0.07 s (range: 0.03–0.23 s) for DIBH and DEBH data and 0.15 s (range: 0–0.43 s) for free‐breathing. Amplitude difference between the normalized CMS and RPM signal during chest and abdominal breathing was within 0.15 for 94.3% of the data points after synchronization. CMS performance was not affected when the subject was clothed.

**Conclusion:**

This novel technology permits sensing of both free‐breathing and breath‐hold respiratory motion. It provides data comparable to the RPM system but without the need for an IR tracking camera in the treatment room or use of reflective markers on the patient.

## 1 INTRODUCTION

External beam radiation therapy (RT) involves the precise delivery of ionizing radiation to predefined locations within the body to kill cancer cells while sparing the surrounding healthy tissue. For many sites, special attention to motion management is required to ensure accurate delivery. One of the most prevalent sources of motion is respiration, and has a prominent effect when treating breast, lung, or abdominal indications. Management of respiratory motion can result in improved targeting accuracy and reduced normal tissue toxicity, and also ameliorates imaging motion artifacts enabling improved tumor visualization and alignment.^1^, ^2^ Common methods of motion management include reduction of motion through abdominal compression, gating to a specific breathing amplitude or phase, and performing defined breath‐hold maneuvers such as deep inspiration breath hold (DIBH) and deep expiration breath hold (DEBH). The DIBH method works by delivering the treatment while the patient holds their breath at the end of a deep inhalation. This method, when used during breast radiation treatment, for example, largely eliminates the breathing motion and pushes the heart further away from the radiation field for left sided breast cancer treatments.^1^, ^3^, ^4^ For thoracic or abdominal indications, DIBH/DEBH acts to stabilize tumor motion, allowing for decreased planning and treatment margins.^1^, ^3^, ^5^ Breath‐hold techniques have shown dosimetric advantages and have become widely used.^6^ In contrast, respiratory gating methods do not eliminate the breathing motion. Rather, they introduce a gating window wherein the radiation beam is delivered during a predefined phase of the breathing cycle. The aforementioned techniques require a continuous monitoring system to ensure the reproducibility of the breathing pattern or breath‐hold position, and the systems employed have traditionally included: laser or optical surface scanning, spirometry, infrared marker tracking, or implanted radiofrequency transponders.^1^, ^2^, ^6^, ^7^, ^8^, ^9^, ^10^, ^11^

Implanted radiofrequency transponders are used for motion management but involve surgical intervention with a chance of major or minor complications, transponder migration, and introduce imaging artifacts.^11^ Spirometric methods work by voluntarily or involuntarily blocking the patient’s breathing. While this may minimize motion, the approach may be limited by a patient's limited respiratory capacity, as well as the equipment costs and patient preparation time.^6^ Infrared tracking devices rely on a limited number of markers placed on the patient’s abdomen or thorax. Markers may be obscured from the IR camera view by patient body habitus, and often need to be placed prior to having complete knowledge of the patient’s breathing habits.^2^, ^10^ While laser‐ or optical‐based surface imaging provides a noncontact three‐dimensional view of the chestwall anatomy, camera placement must allow a nonobstructed view of the patient’s chestwall surface. Accurate surface imaging can be hindered by the position of the gantry/imaging arms and immobilization devices, requires the patient to be fully uncovered throughout the treatment, and may be affected by body hair.^9^ Maintaining a constant and unobstructed view with either reflective marker or surface imaging methods may become more challenging with emerging noncoplanar treatments.^12^, ^13^

In this work, we present the first report of a capacitive‐sensing technology capable of detecting respiratory motion. The technology extends the application of capacitive sensors described previously^14^ in a geometry suitable for sensing motion in various regions of the thoracic or abdominal anatomy. In the development of the prototype device described herein, we aimed to satisfy the requirements of (a) not requiring direct contact with the patient, (b) modularity, that is, a portable device that is moveable and indexable between treatment systems and imaging couch tops, (c) capacity to sense motion through clothing or plastic immobilization devices,^14^ and (d) high temporal frequency (200 Hz) in detecting respiratory motion. We compare this novel technology with the status quo infrared marker tracking approach.

## 2 MATERIALS AND METHODS

### 2.A Capacitive sensing and prototype design

The respiratory monitoring system works by tracking the position of the area of interest, for example, chest wall or abdomen. It can detect the motion of the region of interest (ROI) and provide information used for gating, or determining breath‐hold amplitude. The system is comprised of thin (0.0254 mm) copper conductive sensors mounted on an acrylic horizontal plate above the patient's chest or abdominal area. The human body is naturally electrically conductive,^15^, ^16^, ^17^ therefore placing the copper sensors close to the body forms a capacitor. In its simplest form, the capacitance of a parallel plate system follows Eq. 1. This equation shows that capacitance depends on the distance between the plates (*d*), the area of the capacitor plates (*A*) and the material between the plates which is introduced as permittivity (*ε*). Therefore, capacitance will change as the distance between the sensor and patient changes due to breathing.

(1) $$C=\frac{\varepsilon A}{d}$$

Constant monitoring of the system capacitance allows tracking of the motion of the region of interest. Multiple sensors can be used to track the motion of different regions of the body depending on the clinical needs. Capacitive monitoring of the system can be accomplished by using a capacitive proximity sensor such as MPR121 (Freescale Seminconductor, Inc.). This system has been shown to be stable in linac conditions and under high dose rate photon irradiation.^14^

**Fig. 1:**
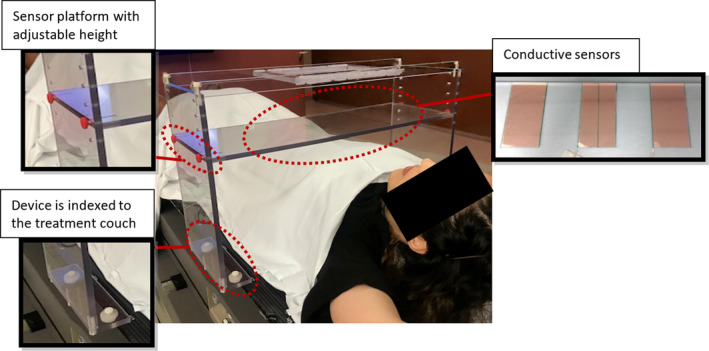
The design of a relocatable respiratory CMS prototype. Three capacitive pads are 5 cm by 10 cm each and mounted 5 cm apart. The current prototype is 60 cm wide, 20 cm deep, and 41 cm high. While the pads are mounted to a Lexan substrate in this implementation, we envisage replacing this material with a minimally attenuating carbon fiber substrate in a clinical version.

**Fig. 2:**
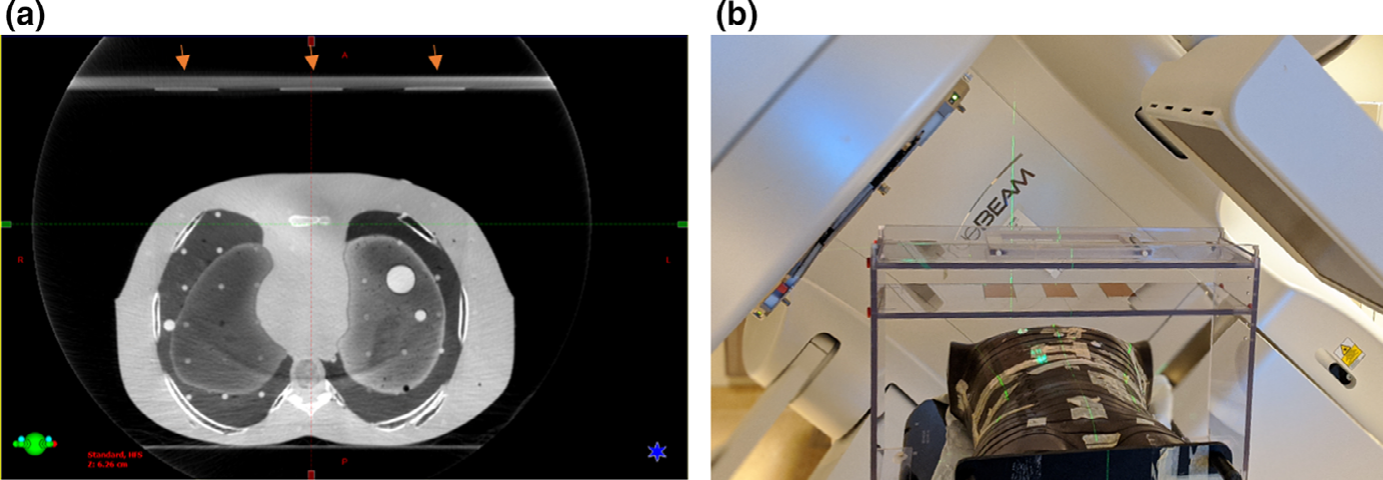
Cone beam CT acquired of the CMS prototype with an anthropomorphic phantom on the linac couch at 95 cm SSD on the Xiphoid position shows no significant image artifacts due to the presence of the copper sensors. The three copper sensors are marked with arrows on the image (a). The setup and gantry clearance are shown in panel (b).

The prototype shown in Fig. 1 is indexed to the treatment couch and can be moved in the cranial–caudal direction to monitor the respiratory motion of different areas such as chest or abdomen. The sensor platform height can be adjusted to accommodate different body types. The thin conductive copper sensors can be placed on the platform in midline or lateral to midline position to monitor the respiratory motion of different regions of interest. The sensors do not come in contact with the patient at any time. While the substrate for the sensors is acrylic in this prototype, in a clinical version of the device, we anticipate that this would be replaced by a rigid, minimally attenuating material such as thin carbon fiber. Additionally, the final design will be optimized to reduce the amount of carbon fiber in the beam, accommodate patient habitus, and reduce gantry clearance issues similar to existing devices such as abdominal compression devices. Further optimization would also provide flexibility by allowing for an arms‐down setup. The prototype measures 60 cm by 20 cm by 41 cm in width, depth, and height, respectively. To ensure clearance with the gantry, the prototype was placed around an anthropomorphic phantom as shown in Fig. 2. The phantom was at 95 cm SSD at xiphoid position and gantry clearance was found to be sufficient during 180 degree rotation. Additionally, CBCT images where acquired to ensure no artifacts are introduced in the presence of the copper sensors (Fig. 2).

**Fig. 3:**
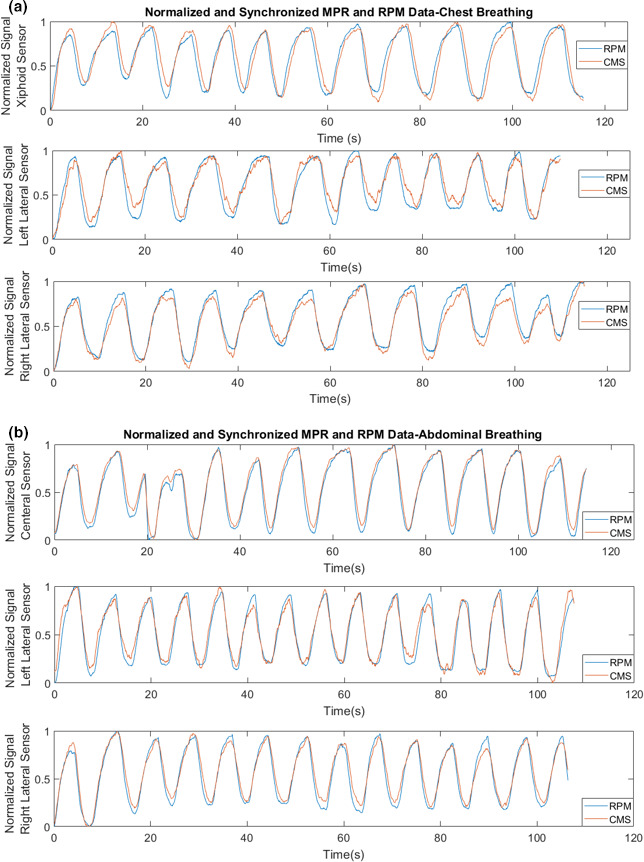
Simultaneous RPM and CMS breathing signal gathered from (a) chest free‐breathing and (b) abdominal free‐breathing for central, left lateral, and right lateral locations. The central sensor was placed on the xiphoid process and 10 cm inferior to the xiphoid process for chest and abdominal breathing, respectively. The lateral sensors were positioned 5 cm lateral to central sensor.

### 2.B Respiratory motion detection

Data collection was performed with the help of a volunteer. The volunteer was positioned supine on the couch, comfortably level, with both arms raised and supported on an indexed breast board (Civco, Orange City Iowa), and made comfortable using a knee wedge. The breast board was centrally indexed to the treatment couch using a standard carbon fiber locating bar (CDR Systems, Alberta, Canada). Experiments were conducted in the CT simulator (Lightspeed 16, GE Healthcare, USA) equipped with standard flat top carbon fiber couch top and real‐time position management system (RPM) (Varian, Palo Alto). The CMS sensors have been shown to be unaffected by the environment of the linear accelerator, and a similar setup and measurement could be achieved there.^14^

The RPM block was setup in contact with the anatomy of interest, and the CMS sensor array was located anterior to the same region. The volunteer was asked to take a deep breath that was used to synchronize both systems in postprocessing. The CMS system was setup to acquire data for 150 s at 200 Hz using in‐house software. Once the CMS data acquisition was concluded, the RPM system was turned off. The CMS data were processed using an exponential weighting method^18^ with a forgetting factor of 0.99 followed by a moving average filter of 10 samples to reduce random noise.

The data were synchronized and normalized to compare the behavior of the two systems. Cross‐correlation analysis was performed to determine any relative time lag (phase shift) between the two datasets. Since the CMS data were acquired at 200 Hz it was downsampled to 30 Hz to match the RPM data and allow cross‐correlation analysis. Amplitude comparison was performed on synchronized and normalized CMS and RPM data during free‐breathing after applying the cross‐correlation shifts.

To investigate the similarity of the measurements from the two systems under different possible clinical conditions, experiments were repeated during chest breathing, belly breathing, deep inhalation, and expiration breath‐hold.

#### 2.B.1 Free‐breathing

Breathing amplitude traces were acquired during chest free‐breathing using CMS and RPM simultaneously, for three ROIs: central, where the central sensor was place on the xiphoid process, and left/right lateral where the sensors were placed 5 cm lateral to the central sensor. An additional set of measurements was acquired during abdominal free‐breathing for central and lateral ROIs which were 10 cm inferior to the chest breathing ROIs.

In each case, the RPM block was affixed to the volunteer's skin and the CMS sensor was positioned above the ROI with a distance of about 8–10 cm from the skin surface. When placing the RPM block in lateral positions, gauze was placed under the block to compensate for the natural curvature of the body and keep the RPM block level and in view of the IR camera.

The volunteer was instructed by a radiation therapist to perform chest breathing by concentrating on sternal rise and fall during inhale/exhale, and belly breathing by concentrating on abdominal rise and fall during inhale/exhale. The volunteer was instructed to maintain even breathing by counting to four each for inhale and exhale. A total of six chest breathing and seven abdominal breathing experiments were performed.

#### 2.B.2 DIBH/DEBH

Breathing traces were acquired during DIBH and DEBH using CMS and RPM simultaneously, for the central region of interest (xiphoid process). In each case the RPM block was affixed to the volunteer’s skin and the CMS sensor was above the ROI with a distance of 8–10 cm from the volunteer.

The volunteer was coached by a radiation therapist to take a slow deep breath to maximally inflate the chest, and to maintain this maneuver for approximately 20–25 s. Additionally, the volunteer was instructed not to arch their back during DIBH/DEBH maneuvers. The volunteer was given time to practice maneuvers and breathing instructions prior to data capture.

#### 2.B.3 Motion detection with obstructed view

Performance of the CMS system was tested with no direct view of the chest. The volunteer was clothed, and the CMS sensor was placed above the xiphoid, again at an 8–10 cm distance from the skin, and data were gathered for three DIBH instances. The RPM system was inoperable in this scenario due to the fact that the reflective block could not be stably positioned on clothing.

### 2.C Effect of sensor‐body separation on signal to noise ratio (SNR)

Another set of volunteer experiments was performed in a Varian Clinac EX accelerator (Varian medical systems) treatment room to determine the effect of different sensor‐body distance on the signal as well as ensure that no additional effects are present as a result of migration to the linac environment. The volunteer was positioned supine on the couch, comfortably level, with both arms raised and supported on an indexed breast board (Civco, Orange City Iowa), and made comfortable using a knee wedge. These experiments monitored abdominal respiration with the sensor placed 10 cm inferior to the xiphoid process in the absence of the RPM block at sensor‐body distances of 3.5, 5.5, and 7.5 cm.

Signal to noise ratio was calculated for the raw data using Eq. (2) below where *A* denotes the amplitude of signal or noise as specified by the subscript.

(2) $$\text{SNR}={\left(\frac{A_{\text{Signal}}}{A_{\text{Noise}}}\right)}^{2}$$

The amplitude of the signal was defined as the average change in acquired signal from exhale to inhale point (breathing amplitude) over the 120 s of data acquisition. The noise was estimated on a 0.005 s rolling window by calculating the signal change between two adjacent data points, averaged over the time series of acquired data (120 s) and rounded. This provides an understanding of the ratio between the range of signal and the amplitude of noise which can be helpful for clinical comparison and decision making.

## 3 RESULTS

### 3.A Capacitive sensing & prototype design

Figure 2 illustrates the gantry clearance with the prototype in place in the linac environment. A snapshot of the acquired CBCT is shown in Fig. 2(b) to show that presence of copper did not introduce image artifacts.

### 3.B Free‐breathing

**Fig. 4:**
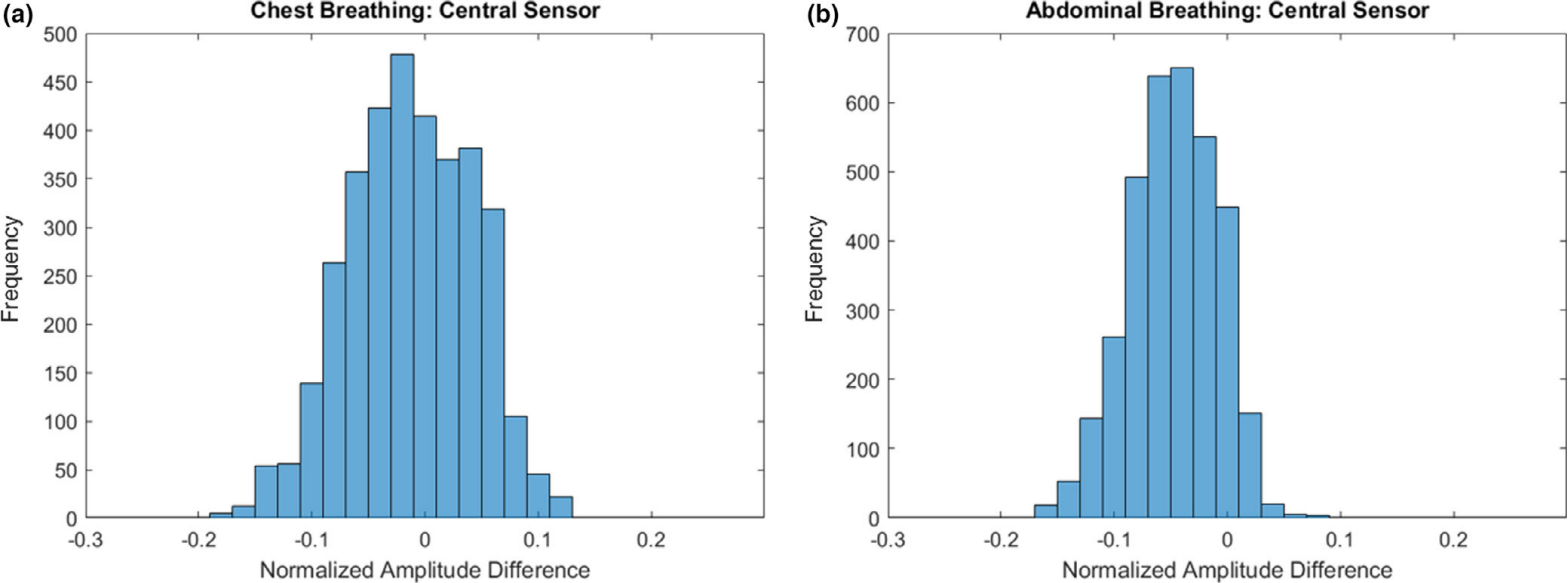
Histogram of normalized amplitude difference between CMS and RPM systems during chest (a) and abdominal (b) breathing. Data were gathered using the central sensor.

Data for the xiphoid sensor trace during chest and abdominal breathing are shown in Fig. 3. Cross‐correlation analysis shows an average absolute lag of 0.16 s (range: 0.03–0.43 s) and 0.14 s (range: 0–0.27 s) for chest and abdominal breathing, respectively. Figure 4 shows a histogram of normalized amplitude differences between the CMS and RPM results after applying the cross‐correlation shift during chest and abdominal breathing. On average, over all 13 chest and abdominal free‐breathing trials, 94.3% of CMS and RPM data points were within 15% of each other. When considering only the central sensor experiments, the value increased to 98.2% and 99.8% for chest and abdominal breathing, respectively.

**Fig. 5:**
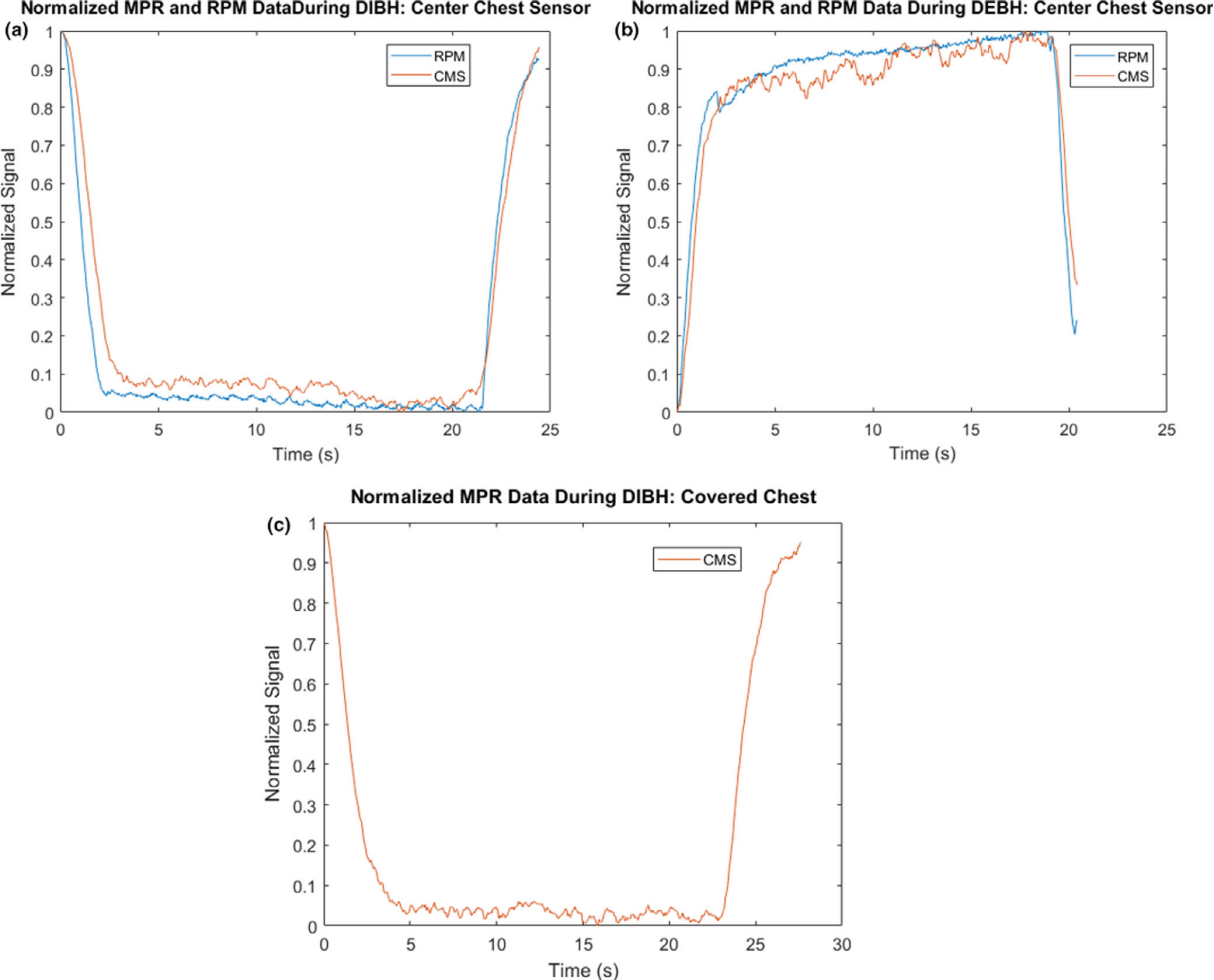
Simultaneous RPM and CMS breathing signal gathered during DIBH (a) and DEBH (b). Sensors were placed over the xiphoid process on bare chest. (c) CMS breathing signal gathered using the central sensor during DIBH with obstructed view of the chest (the volunteer was clothed).

### 3.C Deep inspiration/expiration breath‐hold

Breathing traces for DIBH and DEBH are shown in Fig. 5. The CMS trace during DEBH shows additional noise compared to the RPM data. This is mainly due to the fact that the distance between the chest and sensor plates are maximized during expiration breath‐hold, leading to a decrease in capacitance in accordance with Eq. (1). Cross‐correlation analysis shows an average absolute lag of 0.07 s (range: 0.03–0.23 s) between the CMS and RPM data during DEBH/DIBH maneuvers.

**Fig. 6:**
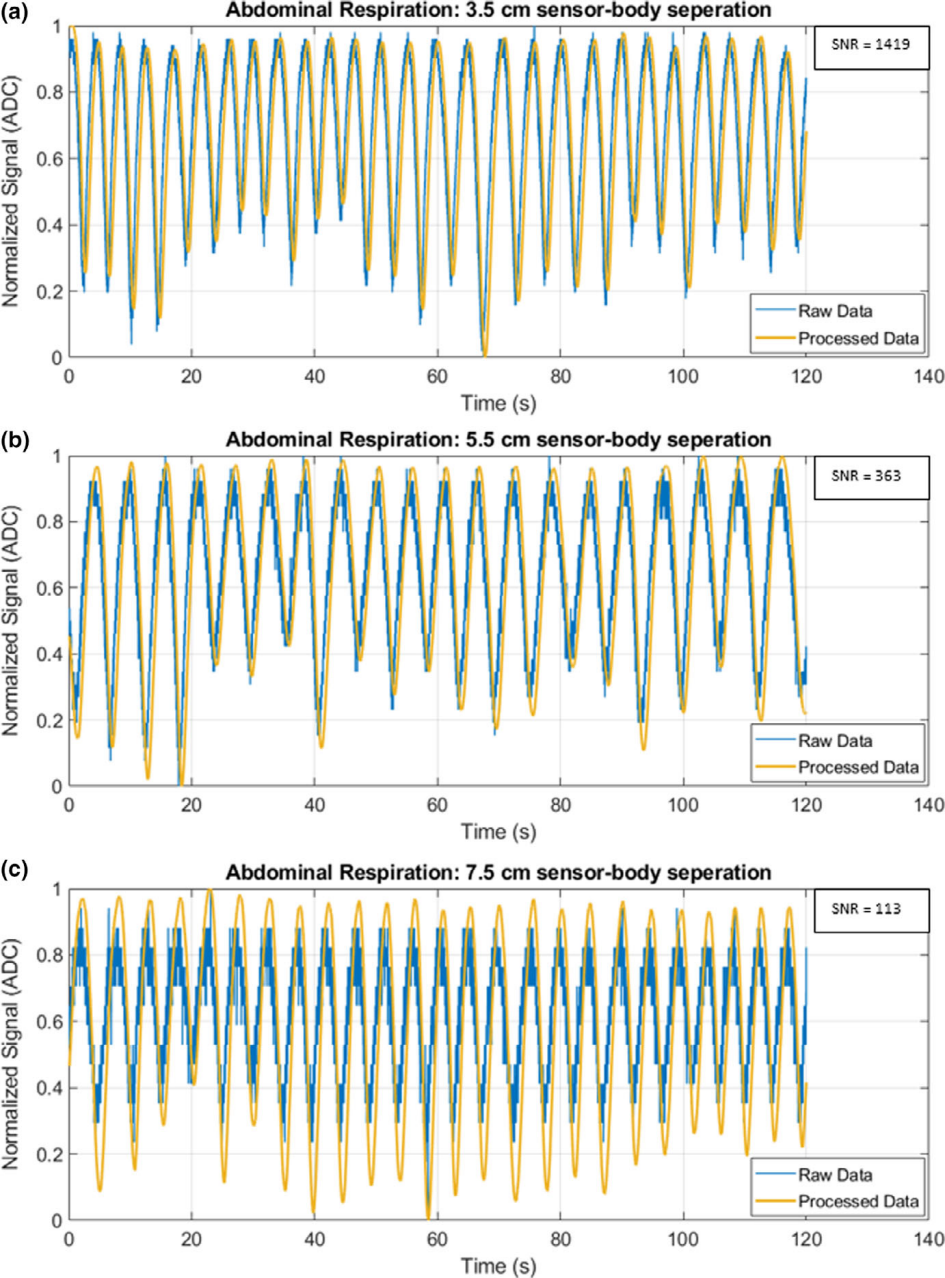
Normalized raw and postprocessed CMS signal acquired during abdominal respiration at different sensor‐body distances. Signal to noise ratio for the raw signal is shown on each graph. The data are acquired at 200 Hz and processed using an exponential weighting method with a forgetting factor of 0.99 followed by a moving average filter of 10 samples (0.05 s) to reduce random noise.

### 3.D Motion detection with obstructed view

Figure 5 shows the results for the DIBH data acquired with the clothed volunteer using CMS, demonstrating that the presence of clothing between the sensor and skin does not pose a limitation for the system.

### 3.E Effect of sensor‐body separation on signal to noise ratio

Figure 6 shows the raw and postprocessed signal at different sensor‐body separation values. SNR ratio for the raw signal is shown on each graph. The SNR values decrease as the sensor‐body distance increases.

## 4 DISCUSSION

This manuscript presents a novel method for noncontact respiratory motion detection. The system provides near real‐time (200 Hz) respiratory motion information during treatment. The system is modular, low cost in comparison to IR or surface monitoring cameras, requires no contact with the patient, and offers the flexibility of using different regions of interest. Additionally, the system does not require unobstructed view of the patient, as it can detect motion through clothes and, was previously shown to detect human motion even within a full thermoplastic mask.^14^ As a result, it can be used in conjunction with different thermoplastic immobilization systems or simple blankets. The sensors and acquisition system have been shown to be stable in the linac environment and under high dose rate photon irradiation.^14^

The modular nature of this device presents several advantages relative to IR or surface imaging camera‐based systems. The device may be coupled reproducibly to a couch top and therefore may be relocated easily between treatment units, CT, PET/CT or angiography platforms. It may also be used outside of these rooms, for example, on a mock‐up of a treatment couch, thereby providing an offline platform for patient education and coaching without tying up expensive capital equipment resources. The device could be produced at comparatively low cost, which is amenable to equipping multiple treatment and imaging rooms. This option is increasingly important, as techniques such as DIBH for treatment of left‐sided breast cancer become more common.^19^

Additionally, while the RPM system detects motion from a single plane, the CMS sensor detects one single capacitance value which is related to the average distance between the sensor and the body over the area of the sensor. As the breathing occurs, the distance between the sensor and body surface varies. The distance averaging resulting from a strip sensor geometry (rather than a point sensor) helps detect the global respiratory motion despite the curvature of the individual’s body.

The system charge/discharge time is 2 µs and draws a low current of 24 µA with the charge/discharge process, which occurs 200 times per second. Considering these variables and the fact that the dielectric material used in this case is air, no significant capacitive leakage or stability issues are observed or expected.

The capacitive system signal relies on the electrical conductivity of the body. This precludes conducting a phantom study, specifically in the chest/abdominal area. In this study, the CMS detection system was used on two volunteers to acquire proof of concept data and to investigate the viability of the design. However, our next step would be a clinical study with a cohort of volunteers in different clinical setup positions. The presented CMS data shows good agreement with the RPM infrared monitoring system (Varian Medical Systems) that has been used for both gating and breath‐hold monitoring in our institution. However, increased noise in signal was observed in case of deep expiration breath‐hold (Fig. 5). This was mainly due to the fact that our CMS sensors were mounted farther away from the chest to allow enough space for the RPM block to be attached to the patient while still ensuring no contact between the block and the CMS sensors occurred during the different breathing stages. This distance was maximized during the deep exhalation breath‐hold and the increase in the chest‐to‐sensor distance resulted in a decrease in capacitance and an increase in noise. This effect could be mitigated during normal operation of the system with the sensors mounted closer to the patient in the absence of the RPM block.

The cross‐correlation analysis shows a small lag between the RPM and CMS systems. The average absolute lag values of 0.07 and 0.15 s were observed for DIBH/DEBH, and free‐breathing, respectively. A contributing factor to the lag is the synchronization process of the RPM and CMS positioning data. That, combined with the mandatory down‐sampling of the high frequency MPR data from 200 to 30 Hz in order to perform the cross‐correlation analysis could explain the sub‐second lag values. However, the respiratory motion with larger amplitude (DIBH\DEBH) shows almost twice the average lag value compared to the respiratory motion with smaller amplitude (free‐breathing). We believe parameters such as the nature of postprocessing of data (our protocol vs what might be used in the RPM software) and the source of the signal acquired (conductive tissue as opposed to the skin motion) could be responsible for the difference.

Amplitude comparison analysis for RPM and CMS central chest and abdominal experiments, showed that on average, 98.2% and 99.8% of all data points were within 15% of one another for chest and abdominal breathing, respectively. The value decreased to 94.3% when averaged over all chest and abdominal respiration experiments for central and lateral sensors. This is possibly due to the fact that, in order to detect the lateral motion using the RPM block, the block is taped in place on a curved section of the body surface and gauze is placed under the block to help compensate for the curvature of the body. This may introduce additional uncertainties in the RPM data due to the reduced stability of the RPM block during respiratory motion.

A comparison of the raw and processed signals shows a slight temporal shift in the processed data. The phase shift was measured at the maxima (end of exhale point) for the cases presented in Fig. 6 and was found to be 6.5°, 8°, and 10° for 3.5, 5.5, and 7.5 cm sensor‐body separation, respectively. As a result, reducing the sensor‐body distance is advised, for example to 4 cm, in clinical conditions. Considering a 40% gating window as an example^20^ (144°), the introduced phase shift at low sensor‐body separation would lead to a small (4.5%) uncertainty.

While this comparison was made to show the similarity between the operation of this technology compared to a clinical status quo (RPM), clinical use of the CMS device would require the acquisition of the breathing trace at the time of CT simulation and during treatment with the CMS device. This would reduce any effects of inter‐device variations in respiratory amplitude detection.

The current prototype provides one dimensional positional information regarding respiratory motion. In concept, the system can be extended to include a larger array of sensors in different axial planes to provide a three‐dimensional mapping of respiratory motion during treatment. The prototype described herein included an acrylic frame for ease of machining and construction. A clinical version will be made of carbon fiber, which is the status quo for clinical accessories in radiation therapy due to its radiotransmissive properties. Additionally, the current design only requires an area of 10 cm by 5 cm for the conductive sensor. This allows for reducing the size of the sensor platform (Figure 1) and optimizing the design to reduce the amount of carbon fiber in the beam, accommodate larger patient habitus, and increase gantry clearance similar to existing devices such as abdominal compression devices.

## 5 CONCLUSION

This work presents a novel noncontact and modular technology for real‐time monitoring of respiratory motion. The current prototype can detect respiratory motion at different regions, providing positional data at 200 Hz readout frequency. The system is minimally intrusive as it does not require unobstructed view of the chest and can provide motion detection for extracranial lesions through fabric, or thermoplastic immobilization material.^14^ Furthermore, the system requires no contact with the patient and is not anchored to a treatment room. This study acts as proof of concept and our next step would be a clinical study with a cohort of volunteers in different clinical setup positions.

## CONFLICT OF INTEREST

Ms. Sadeghi reports grants from Atlantic Canada Opportunities Agency‐ACOA, non‐financial support, and other from Brainlab AG, during the conduct of the study. In addition, Ms. Sadeghi has a provisional patent application pending and a licensing agreement with Brainlab AG. Ms. Moran reports grants from Atlantic Canada Opportunities Agency‐ACOA, non‐financial support, and other from Brainlab AG, during the conduct of the study. Dr. Robar reports grants from ACOA, non‐financial support and other from Brainlab AG, during the conduct of the study, and outside the submitted work. In addition, Dr. Robar has a provisional patent application pending and a licensing agreement with Brainlab AG.
